# Therapists and the Topic of Meaning in Life in Their Encounters With Adolescents With Developmental Trauma: A Qualitative Study

**DOI:** 10.3389/fpsyt.2022.835491

**Published:** 2022-02-18

**Authors:** Kjersti Olstad, Lars Lien, Marja Leonhardt, Torgeir Sørensen, Lars Johan Danbolt

**Affiliations:** ^1^Innlandet Hospital Trust, Brumunddal, Norway; ^2^Norwegian National Advisory Unit on Concurrent Substance Abuse and Mental Health Disorders, Innlandet Hospital, Ottestad, Norway; ^3^Faculty of Health Sciences, VID Specialized University, Sandnes, Norway

**Keywords:** meaning in life, adolescents, therapists, developmental trauma, systematic text condensation

## Abstract

**Background:**

Meaning in life is important to achieve quality of life, psychological well-being and good mental health. Existential issues such as meaning in life have limited attention in mental health care and treatment for children and young people in Norway. People in crisis often ponder existential questions. We find little research on this topic in relation to therapists who work with adolescents with developmental trauma. The purpose of this study was to examine how meaning in life is understood and addressed from the perspectives of therapists working with adolescents struggling with trauma.

**Method:**

The study has a qualitative design, based on focus groups with therapists in mental health care for children and adolescents. The interviews were transcribed and analyzed using systematic text condensation.

**Results:**

Therapists had limited professional experience and competence to address and explore meaning as a topic in therapy. Yet there was interest in the topic and they thought that young people with trauma experience may benefit from the incorporation of meaning perspectives into therapy.

**Conclusion:**

Therapists at a mental health outpatient clinic for children and adolescents found the topic of meaning important but challenging to involve in the treatment of adolescents with developmental trauma. There is a need for more research to enhance understanding of what it means to include meaning as a topic in child and adolescent psychiatry, and what may be the specific benefit and challenges involved.

## Background

Recent years have seen increased interest in the topic of meaning in life in psychology research (1, 2). There is a broad agreement that the experience of meaning in life is important for better quality of life, psychological well-being and good mental health (3–10). Since Holocaust survivor Viktor Frankl (11) wrote about the “will to meaning,” there has been great interest in the importance of meaning in overcoming highly stressful events and increasing motivation to continue living and to receive care and treatment (2, 12–14). Successful attempts have been made to integrate meaning into treatment programs for palliative care patients (1). It is also well-known that patients at risk of suicide need to talk about existential aspects of their challenges (15). A study conducted in a Norwegian child and adolescent mental health outpatient clinic (16) concluded that therapists miss topics of meaning but also find them challenging. How to approach existential topics such as meaning in life when treating adolescents with trauma disorders does not normally form part of the education of therapists and is therefore a focus area of this study. We find various definitions of meaning in life in research (17, 18).

In this study, we draw on Schnell's division into meaningfulness, crises of meaning and sources of meaning (2, 13). To experience life as *meaningful* implies a partly unconscious view of life as coherent, and a sense that life matters and has a direction, together with a feeling of belonging (2, 13). A sense of coherence is fundamental to understanding, managing and finding meaningfulness in one's life (2, 19). A *crisis of meaning* may imply feeling that life is frustratingly empty and meaningless, and that one has no meaning oneself. Such a crisis of meaning can appear as disorientation, a sense of emptiness, and doubts about one's understanding of self and reality. This can be an emotionally painful and paralyzing experience (20). A crisis of meaning presupposes a form of longing for meaning; the person finds it frustrating that life feels empty and meaningless. It is possible to score low on both meaningfulness and crisis of meaning (9, 21), which can be understood as a form of existential indifference (21).

Among the most important *sources of meaning* in life are relationships and meaning something to others, religiosity and spirituality, self-development, creativity and close contact with nature (2, 9).

In this study, we distinguish between the meaning *of* life, which is a philosophical question, and meaning *in* life, which deals with how we experience life. *Meaning in life* is empirical and can be studied as a human experience. It involves the feeling that life is meaningful, the sources that provide meaningfulness and the importance of the experience of meaning for coping with life in particular situations.

It has been shown that crises of meaning can contribute to suicidality in adolescents (20, 22). This indicates a need for more knowledge about understandings and meanings of the topic of meaning in life in encounters with children and young people with experience of trauma. In order to fulfill people's basic need for meaning, it is important to enhance understanding of our existential needs and experiences. Against this background, this article illuminates the topic in collaboration with therapists in Child and Adolescent Mental Health Services (CAMHS) working with adolescents with developmental traumas.

*Developmental traumas* are traumatic experiences in childhood that disrupt the child's development in a number of areas (23), which include neglect and domestic sexual, physical and emotional abuse, as well as frequent separations or violence involving the child's caregivers (24). Nordanger (25) describes developmental trauma as a stressful situation where two negative factors, traumatic stress and poor regulatory support, occur simultaneously. The child is thus subjected to traumatic stress at the same time as a lack of regulatory support from caregivers (25). The term “developmental trauma” reflects the fact that the stress can interfere with the child's development over time. Such traumas can affect the child for many years, often for life (26, 27). For children and adolescents exposed to violence and abuse in the family, their fundamental attachment and security are disrupted (28, 29).

Although meaning in life, including meaningfulness, crises of meaning and sources of meaning, is an extensive area of psychology research (2), which has also been shown to be important for treatment (1), this topic has so far only played a very minor role in the treatment of adolescents with developmental trauma.

The present study aims to explore the experiences of CAMHS therapists of talking to developmentally traumatized adolescents about areas relevant to meaning in life. The study also examines how the therapists reflect on the importance of this topic and their challenges in encounters with developmentally traumatized adolescents in relation to questions of meaning in life.

## Method

### Design and Setting

Given the limited research in this field, we conducted a descriptive qualitative study, exploring the participant's experiences and views in focus group interviews. We found this appropriate for the study of a topic that has previously been little explored (30, 31). We used focus group interviews since they are practical, relevant and useful to enhance knowledge in health service research (32). Focus groups are well suited to a study where the aim is to explore understandings and practices in a group of people with similar experiences. Here, possible conflicts and opposing views will emerge more readily than in individual interviews (31, 33, 34).

This study forms part of a larger project that included two other sub-studies. All three studies focus on the topic of meaning in life in the treatment of young people with developmental trauma, and the studies have collected data from child and adolescent mental health care from a health trust in Norway. In the present study, interviews were conducted with therapists in CAMHS, while in the other two studies, adolescents with developmental trauma in treatment in CAMHS were interviewed.

CAMHS are specialist outpatient clinics for children and adolescents from infancy to the age of 18, offering assessment, diagnostics and treatment of mental health symptoms and conditions. Most patients of CAMHS suffer from various traumatic experiences (35). The varied work of the interdisciplinary therapists in CAMHS includes the identification, assessment, and treatment of different conditions in children and adolescents. In Norway various assessment tools may be used, as well as treatment manuals. The professionals usually consist of physicians, psychologists, nurses, sociologists, and social educators, who generally have clinical specialties and further education. Therapists are divided into teams, usually based on different mental health conditions.

### Participants and Recruitment

The study participants had specific characteristics and knowledge related to our topic. The study was discussed with the management of a relevant CAMHS unit with a view to recruiting therapists. We asked the management for a sample of participants with interdisciplinary expertise, where some had more experience than others. The management approached CAMHS therapists who were mainly involved in trauma treatment. The inclusion criteria were a minimum of 2 years' experience of treating adolescents with developmental trauma and fluency in Norwegian.

Relevant therapists were informed orally and in writing about the study by the manager and the research team; they could then notify the manager of their interest in participation, who would then inform the research team. Ten therapists showed interest in participation. The sample was evenly distributed in terms of participant's clinical experience and profession; there were physicians, psychologists, nurses, social educators, and sociologists with varying degrees of clinical specialization and further education (see Table 1). All were females, with a mean age of 40 years. Females are indicative of the staffing profile, there are few men. In addition, there were only females who said yes to participate. The rationale for grouping older and younger participants was that we thought we could get some different views on the topic because of different work experience.

**Table 1:** Overview of the participants in the focus groups.

**Focus group 1**		**Focus group 2**	
**Age (years)**	**Profession**	**Age (years)**	**Profession**
30	Specialist physician	56	Physician, specialist in child and adolescent psychiatry
23	Social educator	52	Social educator, clinical specialization
24	Psychologist	54	Specialist psychologist
27	Mental health nurse	53	Mental health nurse
25	Sociologist	60	Sociologist, clinical specialization

*Group 1 consisted of younger therapists, while the therapists in group 2 had more experience*.

The therapist's methodological approaches are mainly psychodynamic and cognitive but are not included here as these were not considered relevant to this study. However, they all had experience of working with children and adolescents with trauma.

### Data Collection

The interviews were conducted in October 2019. The sample consisted of two groups with five therapists in each, following methodological guidelines (31). The professions were similar in both groups, but group 2 had more experienced therapists. Each focus group interview lasted for about 90 min. All participants spoke for roughly the same time, thus none of them spoke notably more or less than the others. The participants were engaged in the topic and were all allowed to speak freely and openly. The discussions were specific, to the point and related to the topics introduced through the interview guide. Participants were first asked to reflect on how they as therapists understood the topic of meaning in life, and then asked whether they had experienced that adolescents themselves had raised issues related to meaningfulness during treatment, and if so, whether they could provide examples of this. Participants were also asked whether they themselves had taken the initiative to bring up the topic when talking to adolescents, and if so, how and why, and whether there were particular events or phenomena in the adolescent's history that influenced this. There were no clear differences between the two focus groups regarding how the dialog developed. The interviews were conducted in a pleasant environment (31), in a different location than their workplace.

The second author was the moderator, while the first author took notes. The first author was a former colleague of some of the participants. This meant that the second author was involved from an early stage in the interview process and led the interviews to reduce the impact of bias. This relationship was discussed and reflected on before the interviews and during the analysis. The former collegiate relationship did not seem to have any effect on the results. Audio recordings were made and field notes were taken in order to immediately evaluate interactions and findings (30, 31). All material was then transcribed by the first author.

### Data Analysis

The analysis involved an inductive approach, where empirical data from individual participants helped to shed light on CAMHS therapists experiences of talking to developmentally traumatized adolescents about areas relevant to meaning in life.

The data analysis used systematic text condensation which is useful for this study design (34, 36). This method was especially developed for qualitative health research (37) and consists of four steps. First, we read through the entire material with an open mind to gain a general idea of it and identify preliminary themes. We then read the text closely to identify and code meaning units that represented the participant's experiences and reflections. Next, we summarized and condensed the content of each code group and sorted the different meaning units in each group into subgroups (condensates). During this process, we identified suitable quotes from the material that would illustrate the themes. Finally, an objectified summary description of the content was created, illustrated with individual stories and quotations typical of the topics and findings of the study (37). The research team discussed the results during the four steps. The coding process continued until a high level of agreement was reached in the team (37).

The first author was responsible for the final analysis as it appears in the present text. In the discussion, the findings are discussed in the light of theoretical and psychological concepts of meaning and related to the study context.

### Ethics

The study was assessed and approved by the privacy officer of Innlandet Hospital Trust (#113313). The officer categorized the project as health service research. Participants received written and oral information about the study and signed a consent form prior to the focus group interviews. Participation was voluntary and participants could withdraw from the study at any time without providing a reason.

## Results

The analysis generated three categories. The therapists found that (1) the topic of meaning in life in encounters with developmentally traumatized adolescents was diverse and complex, (2) talking about meaning in life and belief with trauma victims can be challenging, and (3) talking about meaning in life in therapy could help to explore the person's resources and opportunities.

### Meaning in Life in Encounters With Developmentally Traumatized Adolescents Is Diverse and Complex

The concept of meaning was perceived as diverse by the therapists, who gained a deeper understanding of the lives of the adolescents by talking about meaning. Some of the therapists talked about faith and religion as key elements in relation to meaning, and they assumed that meaning could also arouse stronger feelings of guilt and shame in adolescents. Several noted that many adolescents no longer find meaning in school. However, one therapist mentioned her experience that some adolescents could find it very difficult not to feel meaning; instead, they felt doubt and meaninglessness. Several of the therapists stated that adolescents raised thoughts about whether they wanted to live or die, which is not unusual to many of those in therapy for developmental trauma.

Some participants found that several of the adolescents they have in treatment are in a kind of crisis of meaning, and that they are constantly searching for meaning. In connection to this, one of the therapists mentioned adolescents who are keen to find the meaning behind the trauma. The therapists also recalled that some adolescents stated that they felt little or no attachment to their family and had a sense of emptiness and of being left to themselves, and lack of belonging was understood as a factor in relation to meaninglessness. Sometimes meaninglessness was expressed through pondering about lack of meaning when life problems arose, as one said:

“*A bad experience or a crisis makes you ask questions about things. Developmental trauma that you've experienced over time, but also individual events, make you wonder what the meaning of life is.”*

The therapists had observed that adolescents had stated that they felt hopelessness and meaninglessness and then could experience a kind of existential anxiety when topics related to lack of hope and meaning were addressed in therapy, because such topics appeared too large and frightening. On the other hand, the therapists also reflected on the fact that more existential questions, such as knowing who one is in the world, could lead to belief and hope for the future in these young people, and perhaps give new meaning to some of them.

The therapists had different views on what could be considered as sources of meaning for adolescents with developmental trauma. Belonging and relationships were the most important factors mentioned, as one of the therapists said:

“*I think belonging is important, belonging in a group, a group at home, a family group, a choir group, a football group. Those who belong to a group, I think they find life easier than the ones who don't. Much of life is about finding a place to belong. Those who have nothing are in real difficulty. No family, problems at school, no network. Those ones are struggling much more to find out the meaning of things.”*

Having friends and joining clubs and organizations could give these young people a feeling of mastery, a sense of acknowledgment and an experience of meaning something to someone, the therapists stated, and they were concerned that lack of interest and the feeling of alienation of some adolescents could lead to anxiety. Thus, the therapists stated that a sense of coherence in life was important, perhaps especially for this group of adolescents. Routines and everyday activities were emphasized by the therapists for giving meaning in life for traumatized adolescents, such as being outdoors in natural surroundings and being with animals. One participant pointed out that one's family was an important support, while another suggested that family or lack of family could also lead to anxiety and insecurity.

Furthermore, the therapists had the impression that when the adolescents gained more insight into their own situations, this gave many of them greater security and meaning. Importantly, it helped them to believe in the future and that life was worth living.

### Talking About Meaning in Life and Belief With Trauma Victims Can Be Challenging

The therapists stated that several adolescents felt that therapy helped them, and that it could therefore be important for them to talk about meaning in life. This could be an important motivating factor to prevent suicidality. However, some therapists thought that the topic of meaning was too abstract for these adolescents and might be more of a professional approach. They suggested that other terms could be used as an introduction to the topic, such as value, happiness, or interests.

It also emerged from the interviews with the therapists that the topic of meaning did not arise in therapy because the actual traumatic experience sometimes took up so much space that there was no time to talk about other things. One of them said:

“*I think that meaning is kind of connected to something positive and trauma to something negative. The more negative experiences you carry with you in your baggage, the more these may predominate and overshadow what's meaningful and positive. This may mean that something hasn't been worked through. Then that experience will affect the person on a daily basis.”*

When the focus group participants discussed how meaning forms part of treatment, they highlighted faith and religion as topics within meaning. They felt that this was a complex area for these adolescents. Faith could create distance to the closest others and represent a kind of alienation and an obstacle to openness for some of the adolescents who were struggling. One of the therapists said:

“*Faith and religion can be an obstacle to openness about mental illness and the death wish.”*

However, most of the therapists agreed that religion could be important for young people to explore and they also reflected that religion could replace something in families where there is a lack of meaning; in this way, religion becomes a resource. The therapist's opinions varied as to whether faith and one's outlook on life are important for the experience of meaning in young trauma victims. In the focus group discussions, it emerged that some adolescents believe that God is there for some people but not others, and that traumatic experiences make one doubt whether there is a God or whether it is worth believing in anything. One participant mentioned a girl who thought her life experiences were a matter of fate. Another told of adolescents searching for a God or a faith following trauma experiences in order to find peace and strength. One said:

“*Maybe faith is what gives life meaning. Believing in something.”*

The therapists experienced that faith can be for the adolescents both challenging and a resource.

### Talking About Meaning in Life in Therapy Could Help to Explore the Person's Resources and Opportunities

The therapists talked about the importance of finding and exploring the driving force in each individual adolescent and they said that therapy could help these adolescents to better understand their own reactions and feelings and achieve a sense of coherence and meaning in life despite their trauma. One therapist said that several of those she had in therapy did not feel any sense of belonging to their family. She said of one of them:

“*I have a girl in therapy who feels hopelessness in relation to both her parents. Her mother is seriously ill, and she describes her relationship with her father as hopeless. She says she does not feel any attachment anywhere. She's scared when she's with her mother and has a very difficult time when she's with her father. She is on her way to adulthood. She needs belonging and to find meaning if she's to develop. We talk about friendships, good things to support her. We have to constantly find something that means something to her.”*

Some of the therapists specifically mentioned that it was important for these young people to create something, preferably with the therapist. In joint activities, such as drawing or painting, they had found that the adolescents felt acknowledged and gained a sense of mastery. It was also mentioned that the positive therapeutic relationship that some of the adolescents achieve could be an important factor in their lives following the trauma experience. One therapist said:

“*Relationships are very important to me. The relationship between therapist and patient is important. It's important to be seen and acknowledged. When you have a relationship, those things happen.”*

The therapists agreed that if the concept of meaning is to play a greater role in therapy, it may be dependent on a certain level of maturity in the adolescents concerned. They also thought that as therapists they ought to consciously reflect on addressing the topic of meaning.

## Discussion

This study aimed to explore Child and Adolescent Mental Health therapist's experiences of bringing up the topic of meaning in life among adolescents with developmental trauma, and their reflections on the importance or challenges of the topic. The participants had not previously considered meaning in life in treatment in this way. The focus group interviews generated a dynamism that provided the participants with new insights. Their reflections on treatment and their specific experiences from practice could thus shed new light on the importance of involving the topic of meaning in therapy.

In the following, we will address the topics highlighted in the results section and discuss these with reference to research on meaningfulness, crises of meaning and sources of meaning.

The therapists generally thought that an experience of meaning in life is of great importance for developmentally traumatized adolescents. They also said that those who had crises of meaning were seeking meaning and needed to discuss this in therapy. This demonstrated how therapy can be an important setting for exploring what meaning in life involves, and can thus in itself be a source of meaning for these adolescents.

Adolescents commonly become attached to their therapist over time, which helps to give them strength, security and a belief in the future (38). Nevertheless, the therapists described how young people with attachment problems may find the therapeutic relationship difficult, since this is often a consequence of developmental trauma (25, 39–41). All children attach to their caregiver in order to survive (41). As a role model the caregiver can appear safe and corrective. At the same time, a close relationship can feel threatening, since an ambivalent attitude to closeness is part of these adolescent's pathology following their traumas (42, 43).

The therapists emphasized that it was important that the adolescents felt they belonged to something or someone, e.g., having friends and being part of something bigger. These are factors that provide a sense of meaningfulness (2). However, some therapists mentioned that adolescents in this group may tend to isolate themselves from others and avoid social life following their trauma. They may also experience frequent anxiety or depression. To have meaning, i.e., to mean something in the life of others, was described as important in avoiding a feeling of meaninglessness (2). In this connection, the therapists agreed that it was important for several of these adolescents to be with animals, or to create something by painting or drawing. This can provide acknowledgment and a sense of being meaningful, where one's actions have meaning. Other studies have underlined the importance of being with animals (44) and creative activities with adults (45, 46).

The therapists also discussed faith and religion, which they considered both a resource and an inhibiting factor in treatment, as has been seen in other studies on the relationship between religion and mental health (47). For some people, religion can lead to shame and guilt over close relationships in families where there has been abuse combined with a strict religious upbringing (48). Guilt is associated with injustice, and being ashamed implies a feeling that there is something fundamentally wrong with oneself (48). Guilt and shame can be pronounced in adolescents with developmental trauma, and also in adults in long-term therapy for severe mental illness (48–51). However, religion and faith were emphasized as significant factors for some adolescents in their search for security and support, according to the therapists. This concurs with findings that religiosity and spirituality are among the sources of meaning that most strongly correlate with meaningfulness (9). For many, faith and religion can function as resources that help them cope with life crises and serious life events (52).

The therapists found that the adolescents they worked with would sometimes express feelings of emptiness and meaninglessness, which may be understood as forms of a crisis of meaning, namely that life is seen as frustratingly empty and meaningless (2). Research on meaning in life has shown that when people have little sense of coherence in life, feel “my life is meaningless” and that there is no direction or orientation in their lives, or when they lack a feeling of belonging to close relationships, places, education and work, this can create a crisis of meaning that weakens treatment compliance, resilience and the possibility of recovery (13, 20). Several of the traumatized adolescents did not want to live any longer, according to the therapists. Meaning in life has been shown to be important in reducing the risk of suicide (20, 22). The topic has also been brought up successfully in the treatment of other patient groups (1, 15). However, the therapists in this study had little knowledge of crises of meaning and of how they could involve meaning and crises of meaning in therapy.

Excessive focus on the trauma can prevent the therapist from addressing the whole complex picture of the adolescent's problems (53). Therefore, a broader focus in treatment that includes existential themes seems likely to increase these adolescent's hope and expectations and the possibility of a positive treatment outcome (54, 55). However, it can be challenging for therapists to address existential issues in therapy (16). There is also research that shows that young people with trauma may have even less belief in themselves and poorer mental health if meaning and existential topics are discussed with them (56). This shows that competence in this area is essential to enable therapists to work constructively with topics related to meaning in life. The impression from this study is that the therapist's competence in this area is rather general, not based on research or professional development as is the case with much of the other knowledge and skills the therapists possess.

### Strengths and Weaknesses of the Study

Strengths of the study are that it explores a new and under-researched field, and that it employs a clear methodological design. The inclusion of male participants could have been beneficial, but this was not feasible within the framework of the study. Nevertheless, we consider the findings to be new and important for further work in this field.

The role of the first author was deliberately toned down in the interviews, where she only took notes of the discussions. Our analysis of the interviews does not suggest any significant effect of the role of the first author. However, we cannot entirely rule out this possibility.

In order to ensure reliability and validity, the coding of the data was discussed several times in the research team (34). The study's theoretical foundation was derived from both trauma and meaning in life. The objection may be raised that the data are only based on two focus groups, ten therapists, and one treatment unit. However, we considered that this was sufficient, given the topic of the study and the empirical data that formed the basis of the analysis. Although qualitative studies do not permit generalizations, we find that this study has good internal validity. There were no striking differences in the findings between the focus groups with younger and more experienced therapists. One may therefore argue that the findings could be transferable to similar units and environments (57).

## Conclusion

This study has generated knowledge about how a group of therapists in a child and adolescent mental health outpatient clinic found the topic of meaning in life to be important and that addressing this in therapy could help to explore the resources of adolescents and provide coherence in their lives. At the same time, the therapists expressed that issues related to meaning in life were diverse and complex and could be challenging to discuss with adolescents struggling with developmental trauma. Although all participants stated that meaning in life was an important topic to address with these adolescents, this rarely happened in therapy because it was unfamiliar and challenging. The therapists acknowledged that they had not previously viewed the topic of meaning in this way. This suggests a need to enhance knowledge of what meaning in life implies and how it can be used in child and adolescent mental health care. Further research should examine its use in treatment, leading to improved understanding of what it means to include meaning as a topic in CAMHS therapy for developmentally traumatized adolescents and what specific challenges may be involved.

## Data Availability Statement

The original contributions presented in the study are included in the article/supplementary material, further inquiries can be directed to the corresponding author/s.

## Ethics Statement

The study was assessed and approved by the privacy officer of Innlandet Hospital (#113313). Written informed consent to participate in this study was provided by the participants' legal guardian/next of kin.

## Author Contributions

KO and LD designed the study and made the interview guides. KO collected data, conducted interviews, and wrote the manuscript. KO, LL, TS, ML, and LD performed the analysis. All authors contributed to the analysis and participated in revision of the manuscript. All authors have read and approved the final manuscript.

## Funding

This work was financed by Innlandet Hospital, Brumunddal, Norway (#150614).

## Conflict of Interest

The authors declare that the research was conducted in the absence of any commercial or financial relationships that could be construed as a potential conflict of interest.

## Publisher's Note

All claims expressed in this article are solely those of the authors and do not necessarily represent those of their affiliated organizations, or those of the publisher, the editors and the reviewers. Any product that may be evaluated in this article, or claim that may be made by its manufacturer, is not guaranteed or endorsed by the publisher.

## Abbreviations

CAMHS: Child and Adolescent Mental Health Services.
